# Frequency of Leiden Mutation in Newborns with Birth Weight below 1500 g

**DOI:** 10.3390/healthcare10050865

**Published:** 2022-05-06

**Authors:** Jiri Dusek, Lenka Nedvedova, Ondrej Scheinost, Milan Hanzl, Eva Kantorova, Eva Fendrstatova, Radim J. Sram, Hana Kotouckova, Jan Voracek

**Affiliations:** 1 Neonatology Department, Hospital Ceske Budejovice, Bozeny Nemcove 54, 37001 Ceske Budejovice, Czech Republic; lenedvedova@gmail.com (L.N.); hanzl@nemcb.cz (M.H.); fendrstatova.eva@nemcb.cz (E.F.); 2 Faculty of Health and Social Sciences, University of South Bohemia, J. Boreckeho 1167, 37011 Ceske Budejovice, Czech Republic; 3 Laboratory of Molecular Biology and Genetics, Hospital Ceske Budejovice, Bozeny Nemcove 54, 37001 Ceske Budejovice, Czech Republic; osche@nemcb.cz; 4 Department of Genetics, Hospital Ceske Budejovice, Bozeny Nemcove 54, 37001 Ceske Budejovice, Czech Republic; ekant@nemcb.cz; 5 Institute of Experimental Medicine AS CR, Videnska 1083, 14220 Prague, Czech Republic; radim.sram@iem.cas.cz; 6 Department of Mathematics, College of Polytechnics, Tolsteho 16, 58601 Jihlava, Czech Republic; kotouckh@vspj.cz; 7 Department of Technical Studies, College of Polytechnics, Tolsteho 16, 58601 Jihlava, Czech Republic; jan.voracek@vspj.cz

**Keywords:** Leiden mutation, thrombophilia, APC resistance, premature birth

## Abstract

It has been hypothesized that fetal prematurity or Intrauterine Growth Restriction (IUGR) could be related to the presence of factor V of Leiden mutation. This mutation is associated with a higher incidence of pregnancy difficulties that can result in preterm birth. The frequency of Leiden mutation was investigated in the group of newborns with a low birth weight below 1500 g over a six-year period from 2015 to 2020. During this period, 339 newborns were tested, of which 42 newborns with V Leiden mutation (12.4%) were detected. The average of its occurrence frequency in the Czech population was determined as 5.0% based on published studies. In our research, the occurrence of the V Leiden mutation was found significantly higher in newborns under 1500 g. At the same time, we did not demonstrate an increased frequency of births at lower gestational weeks, lower birth weight, or an association with sex in newborns with a positive diagnosis of the Leiden V factor.

## 1. Introduction

The Leiden mutation (V Leiden, LM) is the most common hereditary cause of thrombophilia in the European population, caused by the resistance of activated haemocoagulation factor V to its inhibitor—activated protein C (APC resistance). This mutation was first described in 1993 [1] and named after the discovery site in Leiden, the Netherlands [1]. It probably originated more than 21,000 years ago in the Caucasian population and its occurrence is still strongly influenced by ethnicity and geographic location [2].

The prevalence in the US and European general populations is 3–8% for the factor V Leiden mutation [3].

The prevalence of heterozygotes in the population is reported to be around 5% in the Czech population, while homozygotes are about 1/5000 [4,5,6,7,8]. Factor V Leiden is an autosomal dominant genetic condition that exhibits incomplete penetrance, meaning that not each person who has the mutation will develop the disease. Heterozygosity of this gene increases the lifetime risk of thrombosis 7-fold, while homozygosity (which is rare) increases the risk 20-fold [9].

Studies have shown an increased risk of thromboembolism in pregnancy, the puerperium, oral contraceptives, and individuals with the Leiden Factor V mutation [10,11,12,13].

LM is a major cofactor of Budd–Chiari syndrome developing during pregnancy [14,15].

Circulating microparticles may contribute to the development of thrombosis in carriers of LM [16].

The occurrence of the mutation is associated with the risk of premature births and recurrent spontaneous abortions (especially in the third trimester), while no effect on the incidence of preeclampsia in mothers, foetal hypotrophy, placental dysfunction, foetal IUGR and placental abruption has been demonstrated [17].

The Neonatology Department of the Hospital Ceske Budejovice is one of 12 perinatology centers in the Czech Republic, with a yearly average of 2480 newborns, of which 282 (11.4%) are premature, and 81 (3.3%) are under 1500 g.

## 2. The Aim of the Paper

The determination of the frequency of the mutated factor V Leiden allele in newborns with a birth weight below 1500 g over a six-year period from 2015 to 2020. A higher frequency of this thrombophilia was predicted in this group of newborns.

## 3. Methodology

The frequency of the Leiden mutation was retrospectively determined in neonates born with a birth weight of less than 1500 g. This genetic testing was done with the informed written consent of the mother; neonates of both sexes were examined.

A venous blood sample without clotting was taken from each neonate in a quantity of 0.5–1 mL into Vacutainer tubes with EDTA, stored in the refrigerator and processed within three days of collection. DNA extraction was performed automatically using a Magcore HF16 device and MagCore Genomic DNA Whole Blood Kit (RBCBioscience, Taipei, Taiwan). Mutant and wild-type allele determination was performed using a Real Time PCR kit TaqMan^®^ SNP Genotyping Assay C__11975250 (Thermo Fisher Scientific, Waltham, MA, USA) and LightCycler^®^ 480 II instrument (Roche, Basel, Switzerland).

### Research Limitations

Initially, the investigation process was started with the prior informed consent of the mother; subsequently, a study was created where we focused on the analysis of statistical data to verify the validity of the performed examinations. Ethical approval was requested from the ethics committee of Hospital Ceske Budejovice, a.s.

The feasibility of the study was approved by the Ethics Committee of the Hospital Ceske Budejovice, a.s., on 28 January 2022 under the number 102/22.

## 4. Statistical Evaluation

The statistical program R was used for the statistical evaluation of the study.

Patient cohorts were compared using the Welch Two Sample *t*-test. The χ^2^ test was used to determine the deviation from the Hardy–Weinberg law (*p* > 0.05).

The frequency of occurrence was determined by a one-sample *t*-test.

As a reference value for the prevalence of the Leiden V factor in the Czech population, we used published data [4,7,8]. These studies evaluated a representative sample of 2257 subjects of the Czech population between the years 1997 and 2021. The average of the findings was 5.00% cases with a 95% confidence interval of mean CI = (4.1, 5.9). (Kvasnicka (N = 1527, FVL = 4.5%) [6], Paseka (N = 583, FVL = 6.5%) [8], Riedlova (N = 148, FVL = 4.1%) [3]. N is the number of samples, FVL is the proportion of the mutation found.

## 5. Characteristics of the Studied Cohort

**Figure 1:**
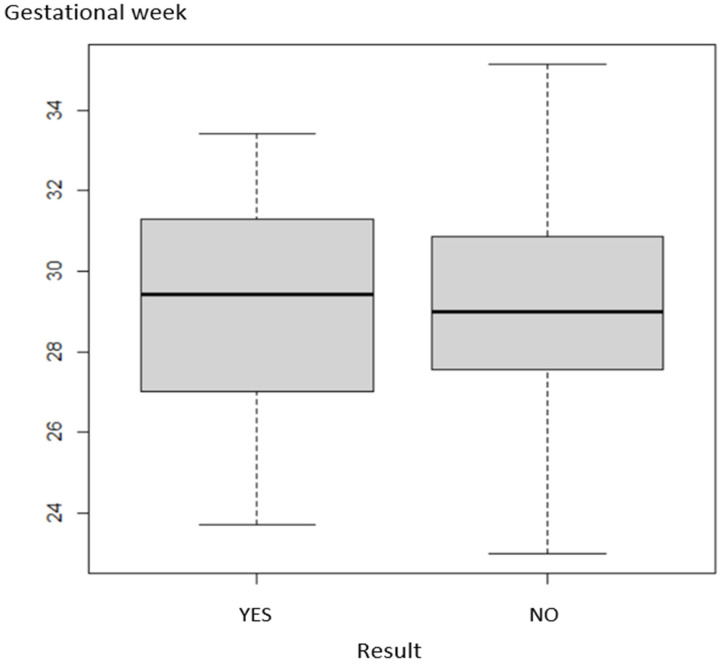
Dependence of positivity on gestational age (*p* = 0.983).

Newborns who met the criterion of <1500 g were born between 23 + 5 gestational weeks and 35 + 1 gestational weeks with birth weights ranging from 470 g to 1500 g inclusive (Figure 1). Of the 483 newborns meeting the criteria, 339 probands were examined. Newborns not included in the study cohort:

- Those whose mothers refused molecular genetic testing for thrombophilic mutations in their offspring;
- Those who were transferred to another lower type of health care facility before blood was drawn for testing, and those who died before reaching 1500 g.

**Figure 2:**
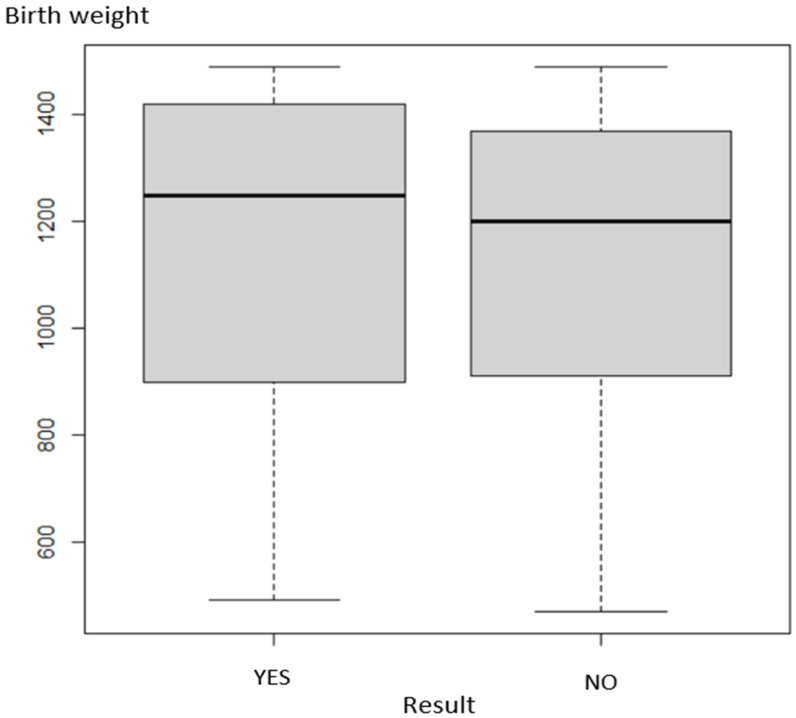
Dependence of positivity on birth weight (*p* = 0.996).

**Figure 3:**
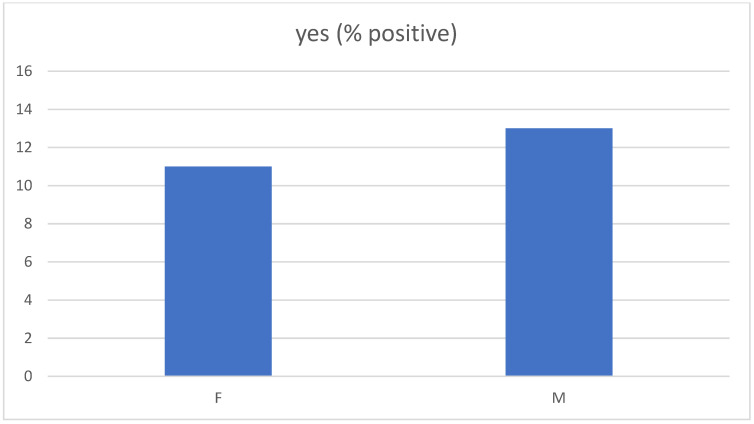
Dependence on sex positivity (*p* = 0.574).

Gestational age, birth weight at birth and sex were assessed for all newborns (Figure 1, Figure 2 and Figure 3). A known positive Leiden mutation was reported in the mothers from medical records (Table 1).

## 6. Results

Data collection over a six-year period from 2015 to 2020 allowed us to evaluate the results of molecular genetic testing for factor V Leiden mutation in selected newborns. During the period in question, a total of 339 newborns were examined for the above-mentioned indications. The V Leiden mutation occurred during the studied 6 years in 42 newborns, which is 12.4% of the studied cohort with a 95% confidence interval of mean CI = (8.9, 15.9) (Table 1).

The homozygote occurred only once, the other were only heterozygotes found.

The expression of the relationship of LM positivity to gestational age is given in Figure 1.

The data showed that the prevalence of factor V Leiden mutation and gestational age at the time of delivery were independent (*p* = 0.983).

The relationship of LM positivity to birth weight is shown in Figure 2. The data indicate that LM was statistically independent of birth weight (*p* = 0.996).

In the sample studied, the representation of boys and girls carrying LM was in the ratio of 13% vs. 11%. In our sample, the prevalence of LM was found to be independent of gender (*p* = 0.574) (Figure 3).

## 7. Discussion

In our study, newborns who had a birth weight below 1500 g were examined for the occurrence of V Leiden mutation. The collection of blood samples from newborns was performed when they reached the weight of 1500 g. It could influence the results due to neonatal mortality. This fact would merit a deeper analysis to see if there is an increased frequency of Leiden mutation in newborns under 1500 g who died in the neonatal period compared to the general population.

The Leiden mutation is a disease that does not pose an immediate threat to newborns. The increased risk occurs later in women using oral contraceptives. Our research shows the risk of that occurrence. A one-sample *t*-test for comparison of our data with the national reference mean illustrates a significant difference between both groups (*p* = 0.0002). Consequently, we concluded that the risk of occurrence of the Leiden mutation in newborns under 1500 g is approximately 2.5 times higher. Certainly, this risk should not be overlooked later in life, especially in women, when other cumulative risks are present. The economic benefit of factor V Leiden screening in women prior to contraceptive use in the Czech Republic would be cost-effective [4]. If it is related to women who weighed less than 1500 g at birth, the risk of serious complications increases even more.

## 8. Conclusions

We focused on the results of thrombophilic mutation V Leiden in newborns with a low birth weight below 1500 g, in whom we could expect an increased incidence of this mutation. We found 12.4% of the incidence over the six-year period where only one was homozygote mutation while the others were heterozygote mutations. We observed a 2.48-fold increase in mean risk compared to the frequency reported in the Czech population. The Leiden mutation was detected at an increased rate in the group of premature newborns with a birth weight below 1500 g.

At the same time, we did not demonstrate an effect of the Leiden mutation on gestational age, birth weight or sex.

## Figures and Tables

**Table 1:** Leiden mutation test results.

Total Newborns	14,949
Total newborns with birth weight below 1500 g/examined	479/339
Leiden mutation in newborns with birth weight below 1500 g (heterozygote/homozygote)	42(41/1)
Percentage of newborns with birth weight below 1500 g with Leiden mutation	12.3
Positive family history (maternal Leiden mutation)	4

## Data Availability

The datasets used and/or analyzed during the current study are available from the corresponding author on reasonable request.
